# Current understanding on micro RNAs and its regulation in response to Mycobacterial infections

**DOI:** 10.1186/1423-0127-20-14

**Published:** 2013-02-28

**Authors:** Pravin Kumar Singh, Ajay Vir Singh, Devendra Singh Chauhan

**Affiliations:** 1 Department of Microbiology & Molecular Biology, National JALMA Institute for Leprosy & Other Mycobacterial Diseases, Tajganj, Agra (UP) Pin- 282001, India

**Keywords:** Micro-RNA, Mycobacterial infection, Tuberculosis, *Mycobacterium tuberculosis*

## Abstract

MicroRNAs (miRNAs) are evolutionarily conserved, naturally abundant, small, regulatory non-coding RNAs that inhibit gene expression at the post-transcriptional level in a sequence-specific manner. Due to involvement in a broad range of biological processes and diseases, miRNAs are now commanding considerable attention. Although much of the focus has been on the role of miRNAs in different types of cancer, recent evidence also points to a critical role of miRNAs in infectious disease, including those of bacterial origin. Now, miRNAs research is exploring rapidly as a new thrust area of biomedical research with relevance to deadly bacterial diseases like Tuberculosis (caused by *Mycobacterium tuberculosis*). The purpose of this review is to highlight the current developments in area of miRNAs regulation in Mycobacterial diseases; and how this might influence the diagnosis, understanding of disease biology, control and management in the future.

## Review

Genomic studies revealed that numerous portions of the human genome do not encode conventional protein-coding genes but encode biologically active non-coding RNA species. With the rapid expansion of small RNA interference techniques over the past decade, it is now clear that many small RNA molecules could regulate gene and protein expression. One class of such small non-coding RNAs is microRNAs (miRNAs), a group of regulatory RNAs of 19–22 nucleotides involved in control of gene expression at the post-transcriptional level [1] thereby acting as RNA interfering (RNAi) molecule. While a well-known RNAi molecule, small interfering RNA (siRNA), is a small RNA that is artificially synthesized, miRNA exists endogenously in the cell. Therefore, miRNAs represents an innate gene-silencing mechanism in our bodies.

miRNAs were first discovered in 1993 while studying *Caenorhabditis elegans*[2]. The first miRNA discovered was lin-4 that was found to play a role in the development through a negative effect on lin-14 expression [2]. After seven years (in 2000), let-7, the second miRNA was discovered, again in the *C. elegans*[3]. In last decade, significant advances have been made in miRNA research leading to the discovery of more than 1,500 miRNAs that have been fully characterized (as per miRBase database viewed in Oct, 2012) and the number is expected to grow in the coming years. Recent studies suggest that miRNAs are involved in regulating cell fate (cell death and proliferation), initiation and progression of human cancer, developmental timing and orchestration of anti-pathogenic responses [4-6].

In view of fact that the miRNAs regulates the expression of a number of genes, the dysregulation miRNAs are being investigated extensively for a number of infectious diseases. Although, early works were focused on the role of miRNAs during viral and parasitic infections [7-9], however in recent past, crucial relevance of miRNAs in the interplay between host and bacteria has been demonstrated [10]. This advancement has now attracted the interest of researcher to dissect the role of miRNAs in most deadly infectious human diseases like Tuberculosis. The genus *Mycobacterium* includes highly pathogenic species *Mycobacterium tuberculosis* (causing tuberculosis) and *Mycobacterium leprae* (causing leprosy) but also opportunists such as *M. avium*, which can also cause disseminated infections in immuno-compromised persons such as AIDS patients [11]. Despite advances in modern medicine and diagnostics, TB remains a major challenge to global public health in the 21^st^ century. Approximately one-third of humanity is infected, but only 5-10% of this population develops active disease, which in 2010 accounted for 8.8 million cases of 1.45 million deaths [12]. Unlike to tuberculosis control, last three decades brought success in leprosy control worldwide but recent emergence of 2,44,796 new cases of leprosy in 2009 challenged both clinicians as well as immunologists, especially in developing countries [13]. Though, interactions between *Mycobacteria* and its environment have been extensively studied, our knowledge at the RNA level is still very limited. Study on deciphering the role of miRNA in mycobacteial diseases, though started very recently, provided some motivating and interesting facts that need to be exploited in future studies for better understanding of disease biology and in designing the efficient control strategies. In this review, we appraise the recent findings on regulation of host miRNAs in response of Mycobacterial infection and underpin the relevance of miRNAs in Tuberculosis.

### Gene structure, biogenesis and basic function of miRNAs

The genesis of functional miRNAs involves a complex multi-enzyme process leading from long precursor molecules into ~22 nt long biologically active RNA molecules. miRNAs are transcribed from their own genes scattered in all chromosomes in humans, except for Y chromosome [14]. Most miRNA genes are located in intergenic regions (intergenic miRNAs) almost >1 kb away from annotated/predicted genes, although some miRNAs were found in intronic regions, within protein coding-genes or in non-coding genes (intronic miRNAs) [15]. Intergenic miRNAs are transcribed as autonomous units with their own promoter/regulatory region, from RNA Polymerase II or III; about a 50% of intergenic miRNAs are found in close proximity to other miRNAs, forming extended clusters which are transcribed as single polycistronic unit [16]. Intronic miRNAs, residing within protein coding genes or non-coding genes, seem to be transcriptionally related to the expression of their host gene and processed in consequence of the spliceosome formation [17-19]. Transcription of intergenic miRNAs, controlled by their own promoter, generates several Kb- long pri-miRNAs with CAP structures and poly(A) tails, which allow its subsequent processing reactions. On the other hand, the transcription of intronic miRNAs underlies the control of their host mRNAs using the same promoter, and involves protein complexes of mRNAs splicing machinery [20].

**Figure 1:**
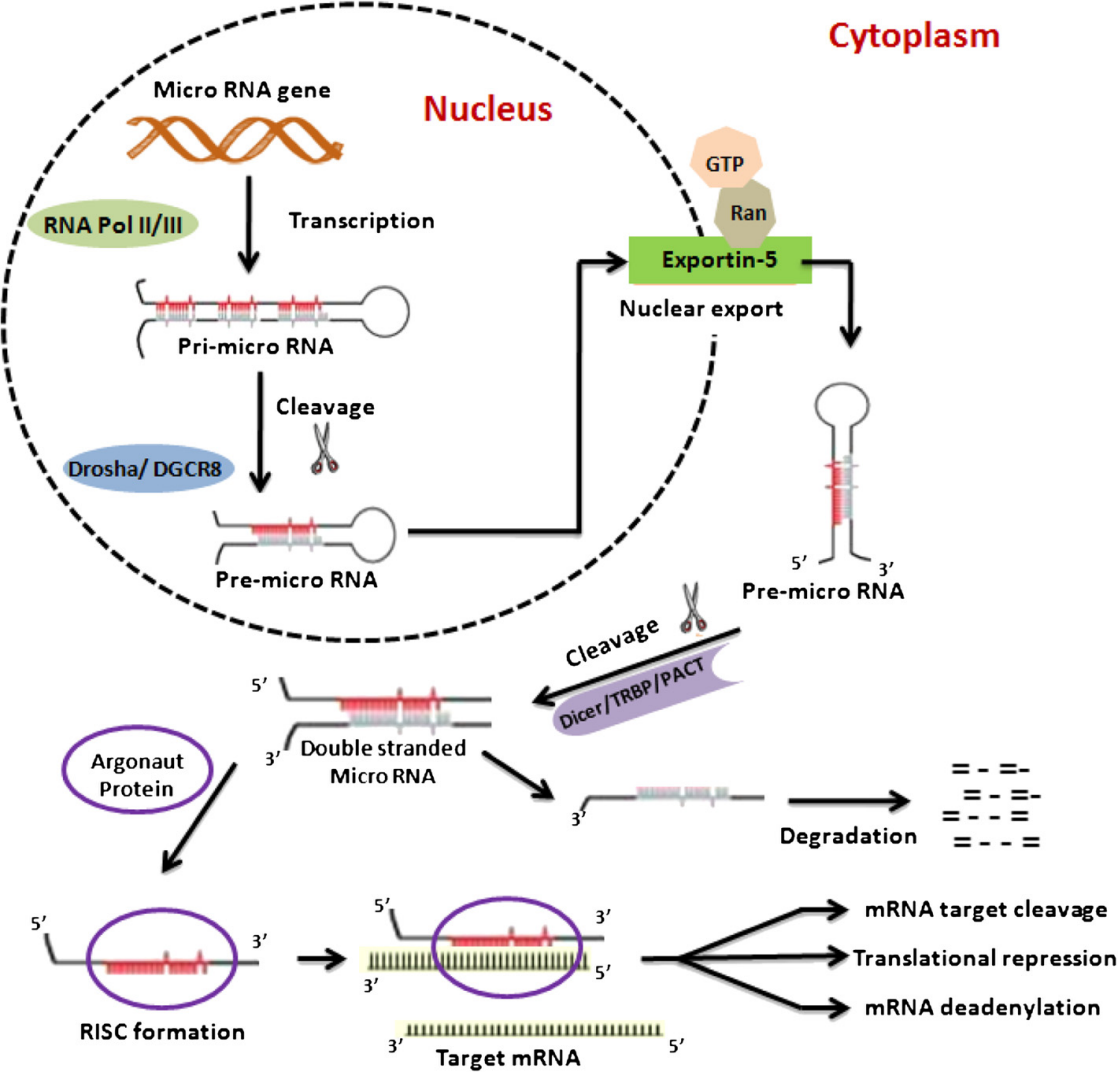
The linear synthetic pathway of micro-RNA biogenesis.

Genes encoding miRNA are generally transcribed in the nucleus by RNA polymerase II (Pol II) into large primary miRNA transcripts (pri-miRNA) (sized >1 kb) that undergo normal further processing i.e. 5^′^-capping 3^′^-polyadenylation or even splicing [21]. However, miRNAs embedded in repetitive elements such as Alus can be transcribed by RNA polymerase III [22]. These RNA molecules form specific hairpin-shaped stem-loop secondary structures and enter a multi-enzyme complex known as a microprocessor to be modified by the RNAse III enzyme Drosha and its co-factor, Pasha. This process leads to the formation of a ~70 nt precursor miRNAs (pre-miRNA) with a 5^′^-phosphate and a 3^′^- 2 nt long overhang [23]. Thereafter, ras-related nuclear protein (RAN-GTP) and exportin 5 mediate the export of pre-miRNAs from nucleus to cytoplasm [24]. Cytoplasmic pre-miRNAs are further processed by another RNAse III enzyme termed Dicer to generate a transient ~22 nt long double stranded miRNA [25]. This duplex is unwounded by helicases into two single strands, one of which is then loaded into the miRNA-associated multi-protein RNA-induced silencing complex (RISC) which includes the Argonaute proteins and induces gene silencing through mRNA cleavage, translational repression or deadenylation. The other strand, passenger strand, of duplex miRNA is degraded [26,27] (Figure 1).

miRNAs are found in plants, animals, and other diverse eukaryotes as well as a number of DNA viruses where it negatively control the expression of target genes. In plants and *C. elegans*, suppression of transcription process was observed [28,29]. This suppression is not a complete shut out, but rather miRNAs are now revealed to fine tune / modulate the rate of mRNA. Though, a few different hypothesis have been given on the action of miRNAs, in general, miRISC with miRNA moves toward the target mRNA, and binds miRNA in the complementary region in the 3^′^ untranslated region (3^′^ UTR) to either terminate the translation or to lead to degradation of the mRNA to interfere with the gene expression [30,31]. Once the miRNA is bound to a completely complementary region of mRNA, like siRNA, mRNA gets degraded. However, miRNA mediated regulation does not require to have a perfect match with its target-binding region. Only, 7-base sequence between 2^nd^ and 8^th^ nucleotide from the 5^′^ end is called “seed region,” and a complete match of the sequence is required. It is believed that the strength of the inhibition varies depending on the sequence but, how it is done at good balance yet to be revealed. A single miRNA may directly affect the expression of hundreds of proteins at once and several miRNAs can also target the same mRNA and result in enhanced translational inhibition [32]. The exact mechanisms of gene repression are still being elucidated, but there is evidence for translational initiation inhibition, translational elongation inhibition, premature translational termination, and co-translational protein degradation [33]. Moreover, these recent studies have introduced a paradigm shift in our understanding of the miRNA biogenesis pathway, which was previously believed to be universal to all miRNAs.

### miRNAs and its relation with bacterial infection

Considering that more than 1,000 miRNAs have been so far annotated in the human genome and that individual miRNAs can have hundreds of targets, it has been predicted that roughly 60% of the human transcriptome may be regulated by miRNAs, although it remains unclear that how many of these are physiologically relevant targets [34]. The complex regulatory role of miRNA adds another layer to an already complex gene regulatory network involved in various biological processes as well as pathogenesis of diseases. There is increasing evidence suggesting that miRNAs play critical roles in many key biological processes, such as cell growth, tissue differentiation, cell proliferation, embryonic development, and apoptosis [35]. The most widely expanding, however, is the disease-related miRNA research based on miRNA expression analysis, probably due to the fact that generalized functional mechanisms were discovered for all miRNA. As per a manually curated, publicly available database (miR2disease.org), more than 150 human diseases (as viewed on Jan, 2013) have been documented to have relationship with miRNAs dysregulation [36]. Although, most of the evidences on involvement of microRNAs in diseases come from cancer research, the dysregulation of microRNAs has been associated with several other diseases viz., neurodegenerative, cardiovascular, pulmonary diseases, liver, kidney, brain and auto-immune diseases. In addition to well established function in physiological and pathological process, miRNAs are increasingly implicated in eukaryotic response to infectious pathogens. This area of research is now gaining the momentum to increase our understanding on complex biology of several infectious diseases.

With respect to infectious diseases, noticeable works was presented on miRNAs regulation in response to viruses and parasites. However, the role of miRNAs in bacterial infection is comparatively less explored area, but recent quantum jump discovered numerous miRNA regulation/ mis-regulation in response to a range of bacterial infections. The first report on role of miRNAs in bacterial infections was documented in plants where Arabidopsis miR-393 contributed to resistance against the extracellular bacteria *Pseudomonas syringae*, presumably by repressing auxin signalling [37]. After this first evidence, the regulations of mammalian miRNAs in response to bacterial infection are increasingly investigated. *Helocobacter pylori* is the main cause of peptic ulceration in human and gastric adenocarcinoma in human [38,39]. Currently, a study showed that *H. pylori* infections alter the expression of oncogenes, tumor suppressor genes and miRNAs [39]. Salmonella has been also found to significantly induce several miRNAs (like miR-155, miR-146a andmiR-21) [40]. Treatment of immune cells with bacterial lipopolysaccharide (LPS) from Salmonella and *Escherichia coli* led to the induction of miR-155, miR-132 and miR-146 expression [41]. The diverse miR-155 response to two subspecies of *Francisella tularensis*, a highly infectious Gram-negative bacterium that causes tularaemia has been reported [42]. Similarly to Gram-negative bacteria, *Listeria monocytogenes* (Gram-positive bacteria), also induce significant changes in the miRNA profile of bone marrow-derived macrophages. Particularly, miR-155, miR-146a, miR-125a-3p/5p, and miR-149 all of which are implicated in regulation of immune related genes, were significantly induced [43]. The rapid progress in miRNA research has provided some concrete evidences on pivotal role of miRNAs in Mycobacterial diseases that are discussed below in detail.

### miRNAs regulation in response to Mycobacterial infection

The exact molecular pathogenesis of tuberculosis and other Mycobacterial diseases is not yet completely understood and this is one of the major hurdle in control of Mycobacterial disease especially Tuberculosis. The slow growth of *Mycobacterium tuberculosis*, coupled with many other quirks, make it a frustrating organism to deal with, while laboratory culture of the other major pathogen, *Mycobacterium leprae* (*M. leprae*), remains an elusive dream. Moreover, the tubercle bacillus has learnt how to defend itself against the existing drug regimens and the emergence of multi and extensive drug-resistant strains of *M. tuberculosis* is now a very real threat to world health. In general, intricate regulation of various eukaryotic/ host genes is crucial in the development of disease, and current understanding of miRNAs has unveiled a new layer of eukaryotic gene expression regulation unravelling several unsolved biological mechanisms in development or disease. This is why, now researchers are giving considerable attention to elucidate the relationship between miRNAs and Mycobacterial infection. Tables 1 and 2 summarize the available reports on the modulation of host miRNAs by some of studied *Mycobacterium* species.

**Table 1:** Regulation of host micor-RNAs in response to Mycobacterial pathogens

**Mycobacterium species**	**Cells/samples and their source**	**Regulated miRNAs**		**Reference**
*Mycobacterium tuberculosis (M.tb)*	Human monocyte derived macrophages	*Up:*	miR-125b	Rajaram et al. [49]
		*Down:*	miR-155	
	PBMCs from TB patients	*Up:*	miR-144*, miR-155, miR-155*	Liu et al. [53]
				Wu et al. [50]
	Serum from TB patients	*Up:*	miR-29a	Fu et al. [45]
	Sputum from TB patients	*Up:*	miR-3179, miR-147, miR-29a	Yi et al. [46]
		*Down:*	miR-19b-2*	Fu et al. [45]
	*M.tb* infected bone marrow derived murine macrophages	*Up:*	miR-155	Kumar et al. [51]
*Mycobacterium leprae*	Skin biopsy from Leprosy patients	*Up:*	miR-21	Liu et al. [54]
	PBMCs from Leprosy patients	*Down:*	mir-181a	Kumar et al. [56]
*Mycobacterium bovis*	Murine T cells	*Down:*	miR29	Ma et al. [48]
*Mycobacterium avium*	Human monocyte derived macrophages	*Up:*	miR-155, miR-146a/b, miR-886-5p, let-7e, let-7i, miR-29a	Sharbati et al. [47]
		*Down:*	miR-20a, miR-191, miR-378, miR30c, miR-423-5p, miR-374a, miR-185, miR-768-5p	
*Mycobacterium smegmatis*	Human monocyte derived macrophages	*Up:*	miR-155	Rajaram et al. [49]

**Table 2:** Identified micro-RNAs mediated modulation of host response during Mycobacterial infection/ stimulation

**Micro-RNAs**	**Identified regulation mechanism**	**Reference**
miR-29	Suppress the IFN-γ production by targeting IFN-γ mRNA	Ma et al. [48]
miR-29 and Let-7e	Inhibit the apoptosis by caspases 3 and 7 (major effector caspases)	Sharbati et al. [47]
miR 125b	Block the TNF-α biosynthesis	Rajaram et al. [49]
miR-155	Enhance the TNF-α biosynthesis	Rajaram et al. [49]
miR-155	Repress Bach1 and SHIP1 to support the dormancy and survival of *M. tuberculosis* in host	Kumar et al. [51]
miR-99b	Negatively regulate the production of proinflammatory cytokine that leads to reduced growth of *M. tuberculosis*	Singh et al, [50]

miRNAs have been found in tissues and also in serum and plasma, and other body fluids, in a stable form that is protected from endogenous RNase activity (in association with RISC, either free in blood or in exosomes (endosome-derived organelles)). The potential for the use of these circulating miRNAs as biomarkers of disease and as targets of therapeutics is promising for various diseases especially cancer [44]. In view of potential significance of circulating miRNAs, the first success in clarification that miRNAs takes role in pulmonary tuberculosis pathogenesis was published in 2011 [45] in which the relationship between circulating serum miRNAs and active pulmonary tuberculosis was investigated by micro-array based expression profiling method. Among 92 miRNAs detected significantly, 59 miRNAs were downregulated and 33 miRNAs were upregulated in the TB serum compared to their levels in the control serum. Interestingly, on validating the micro-array results with real time PCR, differentially increased expression of miR-29a and miR-93* was found not only in the serum but also in the sputum of patients with active pulmonary tuberculosis as compared to healthy controls [45]. After this first description, subsequently, genome wide miRNA expression in sputum supernatant of patients with active pulmonary tuberculosis was also delineated in recent past [46]. In this study, a total of 95 miRNAs were found to be expressed differentially by microarray and miR-3179, miR-147 overexpressed and miR-19b-2* suppressed in TB patient group compared with controls as observed in the validation cohort by real time PCR.

In 2011, Sharbati and co-workers [47] demonstrated how distinct mycobacteria could manipulate host cell response by studying the regulatory network of potential interactions between miRNAs and mRNAs. The human macrophages infected with *M. avium* revealed that many of the differentially regulated miRNAs showed decreased expression (e.g., miR-20a, miR-191, miR-378, miR-185), whereas miR-155, miR-146a/b, miR-886-5p, miR-29a and let-7e were induced upon mycobacteria infection. The integrated analysis of miRNA and mRNA expression as well as target prediction and reporter assays identified caspases 3 and 7, major effector caspases essential in the triggering of apoptosis, as targets of let-7e and miR-29a, respectively, thus showing that inhibition of apoptosis after mycobacterial infection is controlled by miRNAs. The pivotal role of miR-29 was also demonstrated in T cells of mice infected with *Listeria monocytogenes* or *Mycobacterium bovis bacillus* Calmette-Guerin (BCG) [48]. However, in contrary to above observed induction of miR-29a, down-regulated expression of miR-29 in IFN-γ-producing natural killer cells, CD4^+^ T cells, and CD8^+^ T cells was observed. Interestingly, it was demonstrated that miR-29 directly target IFN- γ mRNA and suppress the production of IFN-γ [48].

Additionally, differential miRNA regulation was also reported recently in response to high virulent Mycobacteria (*M. tuberculosis*) as compared to low virulent (*M. smegmatis*) in infected human macrophages [49]. Macrophages incubated with surface molecule (lipomannan) of *M.tuberculosis* and live induce high miR-125b expression and low miR-155 expression with correspondingly low TNF production. Whereas, lipomannan of *M. smegmatis* and live induce high miR-155 expression and low miR-125b expression with high TNF production. The differential induction of TNF-α biosynthesis by the two mycobacteria species may be understood as; miR-125b directly targets the TNF-α mRNA, while miR-155 probably indirectly enhances TNF-α production by increasing TNF-α mRNA half-life and translation [41]. Therefore, high levels of miR-125b induced by *M. tuberculosis*, leads to the blocking of TNF biosynthesis, thereby allowing *M. tuberculosis* to subvert host immunity and potentially increase its virulence. In a very latest study, miR-99b mediated TNF-α modulation was also demonstrated that affects the bacterial growth in *M. tuberculosis*-infected dendritic cells [50]. Since, knockdown of miR-99b in dendritic cells associated with enhanced production of proinflammatory cytokines and significantly reduced bacterial growth, it will be interesting to explore further the potential of miR-99b to be used as therapeutics/ biomarker for tuberculosis. In a different study, higher expression of miR-155 and miR-155* was observed in stimulated PBMCs (with tuberculin; antigen mixture of standard H37Rv strain of *M.tuberculosis*) of active tuberculosis patients as compared to un-stimulated [51]. Therefore, tuberculin-induced increased expression of miR-155 and miR-155* explored its future exploitation as a diagnostic biomarker. However PPD is not considered a reliable stimulator, and it may trigger false-positive results in BCG-vaccinated or tuberculin-positive individuals. Very recently, specific secretory *M. tuberculosis* antigen, ESAT-6 was also found to play a key role in miR-155 induction and its subsequent effects on Bach1 and SHIP1 repression [52]. It is known that Bach1 is a transcriptional repressor of heme oxygenase-1 (HO-1; a documented activator of the *M.tuberculosis* dormancy), whereas SHIP1 inhibits the activation of the serine/threonine kinase AKT (required for *M.tuberculosis* survival). Therefore, it seems that miR-155 regulation is critical in modulation of host innate immunity and for protection against *M. tuberculosis* infection. *M. bovis* BCG was also found to trigger Toll-like receptor 2 (TLR2)-dependent miR-155 expression that takes part in mediating the apoptosis of macrophages through complex signaling pathways [53]. Thus it again suggested the importance of miR-155 and its role in orchestrating cellular reprogramming in response to Mycobacterial infection.

In addition to miR-155, the role of miR-144* precursor in modulation of anti-TB immunity through modification of cytokine production and cell proliferation of T cells was also demonstrated [54]. Besides, overexpression of miR-144* precursor in PBMCs of pulmonary tuberculosis cases, transfection of T cells with miR-144* precursor demonstrated that miR-144* might inhibit TNF-α and IFN-γ production and T cell proliferation.

Recently, miRNA expression in leprosy skin lesions was also investigated in which 13 miRNAs were found to be expressed differentially in the lesions of human subjects with progressive lepromatous (L-lep) versus the self-limited tuberculoid (T-lep) disease [55]. mir-21, most differentially expressed miRNA in L-lep lesions was also overexpressed in *M.leprae* infected monocytes. Notably, it was also experimentally demonstrated that mir-21 inhibits the expression of the genes encoding two vitamin D–dependent antimicrobial peptides, CAMP and DEFB4A probably by direct downregualation of Toll-like receptor 2/1 heterodimer (TLR2/1)-induced CYP27B1 and IL1B expression as well as indirectly upregulating interleukin-10 (IL-10). It is again noteworthy that knockdown of mir-21 in *M. leprae*-infected monocytes enhances the expression of CAMP and DEFB4A and restore TLR2/1-mediated antimicrobial activity against *M. leprae*. Therefore, study strongly suggests that mir-21 can potentially downregulate host defense genes (to escape from vitamin D–dependent antimicrobial pathway) in leprosy. NF-kB activation mediated upregulation of miR-21 in response to Bacillus Calmette-Guerin (BCG) vaccination was also delineated in recent past [56]. Additionally, the role of miR-21 in suppression of IL-12 production (by targeting IL-12p35) and inducted dendritic cell apoptosis (by targeting Bcl-2) was mark out in BCG-vaccinated bone marrow derived macrophages. Therefore, this study again strengthened the role of miR-21 in fine-tuning of the anti-mycobacterial response in general and regulating the efficacy of BCG vaccination in particular. Besides the critical role of miR-21 in leprosy, miR-181a is another important RNA molecule which down-regulated expression may be associated with leprosy progression [57]. Furthermore, it was suggested that miR181a may contribute to overexpression of SHP2 that lead to T cell hyporesponsiveness during leprosy progression. Keeping all the above recent finding in notion, it is now clear that Mycobacteria can induce miRNA expression in immune cells and also miRNA plays important role both in progression as well as protection of Mycobacterial diseases.

#### Role of miRNAs as biomarker and therapeutics in Mycobacterial diseases

miRNA expression profiling is of increasing importance as it’s exploitation in development of reliable diagnostic and prognostic biomarkers. In last few years, studies especially on role of miRNAs in different types of cancer have shown that miRNA expression profiles may classify the tumors and also it may potentially be used as biomarker for diagnosis and disease progression [58,59]. Encouraged with cancer studies, the role of miRNAs in infectious diseases including bacterial diseases was investigated. Although, some tantalizing evidences has been put forward on the possible role of miRNAs as biomarker for bacterial diseases including Mycobacterial infection, continued and extensive works however warranted in light of the marked global health impact of tuberculosis and problems associated with its diagnosis. As discussed in previous section, differential miRNA levels have been found in PBMCs [51,54], serum [45] and sputum [46] of TB patients as compared with healthy control. Of the various miRNAs, miR-29a, miR-155, miR-155*, miR-125b, miR-3179a and miR-147 may be of potential biomarker for diagnosis of tuberculosis (Table 1). Although, these miRNAs biomarkers have been identified by some independent groups but still it could not be validated adequately. Moreover, if we consider that these have potential to discriminate active and / or latent tuberculosis from healthy individuals, the question remains whether these gene expression signatures are specific for TB or shared, at least in part, with diseases of similar pathology but distinct etiology. This important issue was investigated in a recent study, in which whole blood miRNAs signatures of tuberculosis was compared with sarcoidosis that is known to have similar pathology [60]. This study showed significant differences in expression between healthy and diseased individuals, however, both TB and sarcoidosis revealed highly similar miRNAs profiles. The similarity of miRNAs profile may be due to the fact that miRNAs are primarily responsible for fine tuning of responses rather than on/off switch signals [61]. Therefore, on the basis of current understanding on this aspect, future works need on identifying the biomarkers by not only focusing on differential expression but also expression in diseases with similar pathology or diseases caused by closely related pathogens (like *Mycobacterium* species).

Besides use as a biomarker, another translational application of miRNAs has been explored as therapeutics. There are two main strategies for developing drugs targeting miRNA: (1) by suppressing the disease-specific miRNAs whose expression are increased in diseases and (2) by supplementing miRNA whose expression decreased. Tremendous efforts and progress are being made for the development of miRNAs based drugs especially for cancer [62] and Hepatitis C virus infection [63]. The differentially expressed miRNAs found in response to Mycobacterial infection (Table 1) may have potential as future therapeutics however, no clinical trials and progress in this line have been made so far.

#### Future direction: Expectation and limitation

Despite of several advancement and rigorous research of last several decades, tuberculosis remains most deadly, major threat to mankind and an enigmatic infectious disease. Of the various research efforts in diverse directions for the control of tuberculosis, recently identified involvement of miRNA in mycobacterial infection has also nourished the hopes for better understanding of pathogenesis, developing new class of sensitive and accurate diagnostic and prognostic biomarkers and possible new therapeutics for tuberculosis. But currently this area is in its infancy and demanding more attention and continued works towards understanding the complex regulation of miRNA regulation in tuberculosis and further its exploitation to add the rational design for the effective control and management of tuberculosis. The accurate and rapid diagnosis as well as ability to monitor the treatment response is very crucial for effective control and management of tuberculosis. Although few miRNAs has been identified having ability to differentially diagnose the active TB and latent TB from healthy individuals [45,64] but whether such biomarkers are of TB or shared by other diseases is yet not clear. Therefore, along with developing reliable miRNAs based biomarkers, future works are needed to discover biomarkers for the prediction of relapse, sterilizing activity and treatment response on account to provide batter treatment as well as to facilitate the testing of new drugs.

Recent landmark *in- vitro* and *in-vivo* studies in Mycobacterial diseases showed that miRNA species, regulating immune modulatory genes directly or indirectly, can affect the downstream effectors of an innate immune-triggered antimicrobial pathway [48,54] and thereby contributing in development of disease. This knowledge may have implications for the development and improvement of future approaches for the prevention and therapy of tuberculosis. Since the identification of disease-specific miRNAs that cause the onset or exacerbation will lead to intellectual property as well as drug development, great efforts are made to research and develop them worldwide especially in cancer and a few other diseases. Although there are some successes and few miRNAs targeting drugs are currently under different phase of clinical trials, still suitable delivery system for miRNA drug and it’s sustaining potency inside the body still need considerable attentions in future research. Recently, potential target sites of known human miRNAs were identified in *M. tuberculosis* genome by *in-silico* prediction method [65]. However, due to limitations of current miRNA target prediction programs, experimental validation of predicted miRNA targets is necessary to confirm. If host cell miRNAs are proven to play a role in regulating the intricate networks involved in human-*M.tuberculosis* interaction, it will open up a new area in both miRNA and *M. tuberculosis* research.

The limitation to applicability of miRNAs as biomarker and therapeutics reflected by the very basic and still unresolved question as to whether the increase or decrease of miRNA expressions actually cause the disease or if they are a consequence of the disease. Additionally, it is known that miRNAs contribute in various biological processes and single miRNA may modulate the expression of hundreds of genes therefore administration of miRNA as drug to cure any targeted disease, may lead to unwanted gene silence. Therefore, the above challenges should be kept in notion while taking the initiative for the development of miRNAs targeted/ based biomarkers or therapeutics for tuberculosis or other diseases. Overall, in future greater emphasize on basic research is needed simultaneous with research for translational application of miRNAs in mycobacterial diseases, especially in tuberculosis.

## Conclusions

miRNAs represent a relatively young field of basic biological and translational research into new and innovative therapeutic applications. Tantalizing preliminary evidence, miRNAs have altered expression and are able to modulate the host antibacterial pathways in response to Mycobacterial infection, have created new opportunities in tuberculosis research. Since research on similar line begun only recently, the future research on identification and detail understanding of how host cell miRNAs regulate Mycobacterial infection will be of exquisite importance in view of development of novel biomarkers and therapeutics. The rapid advancement and explosion in miRNA research nourishes our hope for a giant leap in better diagnosis and treatment of infectious diseases including tuberculosis in future.

## Competing interests

The authors declare that they have no competing interests.

## Authors’ contributions

PKS and DSC designed the concept. PKS and AVS collected information, and prepared the manuscript and figures. PKS and DSC wrote the final manuscript. All authors read and approved the final manuscript.

## References

[B1] Bartel DP MicroRNAs: genomics, biogenesis, mechanism, and function Cell 2004 116 2 281 297 10.1016/S0092-8674(04)00045-5 14744438

[B2] Lee RC Feinbaum RL Ambros V The *C. elegans* heterochronic gene lin-4 encodes small RNAs with antisense complementarity to lin-14 Cell 1993 75 5 843 854 10.1016/0092-8674(93)90529-Y 8252621

[B3] Reinhart BJ Slack FJ Basson M Pasquinelli AE Bettinger JC Rougvie AE Horvitz HR Ruvkun G The 21-nucleotide let-7 RNA regulates developmental timing in *Caenorhabditis elegans* Nature 2000 403 6772 901 906 10.1038/35002607 10706289

[B4] Ambros V The functions of animal microRNAs Nature 2004 431 7006 350 355 10.1038/nature02871 15372042

[B5] Voinnet O Induction and suppression of RNA silencing: insights from viral infections Nat Rev Genet 2005 6 3 206 220 10.1038/nrg1555 15703763

[B6] Taganov KD Boldin MP Baltimore D MicroRNAs and immunity: tiny players in a big field Immunity 2007 26 2 133 137 10.1016/j.immuni.2007.02.005 17307699

[B7] Ding SW Voinnet O Antiviral immunity directed by small RNAs Cell 2007 130 3 413 426 10.1016/j.cell.2007.07.039 17693253 PMC2703654

[B8] Cullen BR Viruses and microRNAs: RISCy interactions with serious consequences Genes Dev 2011 25 18 1881 1894 10.1101/gad.17352611 21896651 PMC3185961

[B9] Hakimi MA Cannella D Apicomplexan parasites and subversion of the host cell microRNA pathway Trends Parasitol 2011 27 11 481 486 10.1016/j.pt.2011.07.001 21840260

[B10] Eulalio A Schulte LN Vogel J The mammalian microRNA response to bacterial infections RNA Biol 2012 9 10 742 750 22664920 10.4161/rna.20018

[B11] Corti M Palmero D *Mycobacterium avium* complex infection in HIV/AIDS patients Expert Rev Anti Infect Ther 2008 6 3 351 363 10.1586/14787210.6.3.351 18588499

[B12] World Health Organization Global tuberculosis control report 2011 Geneva: WHO

[B13] World Health Organization Global Tuberculosis Control report. Epidemiology, Strategy, Financing 2009 Geneva: WHO

[B14] Rodriguez A Griffiths-Jones S Ashurst JL Bradley A Identification of mammalian microRNA host genes and transcription units Genome Res 2004 14 10A 1902 1910 10.1101/gr.2722704 15364901 PMC524413

[B15] Kim NV Jin-Wu N Genomics of microRNA Trends Genet 2006 22 3 165 173 10.1016/j.tig.2006.01.003 16446010

[B16] Lee Y Jeon K Lee JT Kim S Kim VN MicroRNA maturation: Stepwise processing and subcellular localization EMBO J 2002 21 17 4663 4670 10.1093/emboj/cdf476 12198168 PMC126204

[B17] Lagos-Quintana M Rauhut R Lendeckel W Tuschl T Identification of novel genes coding for small expressed RNAs Science 2001 294 5543 853 858 10.1126/science.1064921 11679670

[B18] Mourelatos Z Dostie J Paushkin S Sharma A Charroux B Abel L Rappsilber J Mann M Dreyfuss G A novel class of ribonucleo proteins containing numerous microRNAs Genes Dev 2002 16 6 720 728 10.1101/gad.974702 11914277 PMC155365

[B19] Lau NC Lim LP Weinstein EG Bartel DP An abundant class of tiny RNAs with probable regulatory roles in *Caenorhabditis elegans* Science 2001 294 5543 858 862 10.1126/science.1065062 11679671

[B20] Lee Y Kim M Han J Yeom KH Lee S Baek SH Kim VN MicroRNA genes are transcribed by RNA polymerase II EMBO J 2004 23 20 4051 4060 10.1038/sj.emboj.7600385 15372072 PMC524334

[B21] Cai X Hagedorn CH Cullen BR Human microRNAs are processed from capped, polyadenylated transcripts that can also function as mRNAs RNA 2004 10 12 1957 1966 10.1261/rna.7135204 15525708 PMC1370684

[B22] Borchert GM Lanier W Davidson BL RNA polymerase III transcribes human microRNAs Nat Struct Mol Biol 2006 13 12 1097 1101 10.1038/nsmb1167 17099701

[B23] Denli AM Tops BB Plasterk RH Ketting RF Hannon GJ Processing of primary microRNAs by the Microprocessor complex Nature 2004 432 7014 231 235 10.1038/nature03049 15531879

[B24] Bohnsack MT Czaplinski K Gorlich D Exportin 5 is a RanGTP-dependent dsRNA-binding protein that mediates nuclear export of pre-miRNAs RNA 2004 10 2 185 191 10.1261/rna.5167604 14730017 PMC1370530

[B25] Hutvagner G McLachlan J Pasquinelli AE Balint E Tuschl T Zamore PD A cellular function for the RNA-interference enzyme Dicer in the maturation of the let-7 small temporal RNA Science 2001 293 5531 834 838 10.1126/science.1062961 11452083

[B26] Schwarz DS Hutvagner G Du T Xu Z Aronin N Zamore PD Asymmetry in the assembly of the RNAi enzyme complex Cell 2003 115 2 199 208 10.1016/S0092-8674(03)00759-1 14567917

[B27] Winter J Jung S Keller S Gregory RI Diederichs S Many roads to maturity: microRNA biogenesis pathways and their regulation Nature Cell Bio 2009 11 3 208 234 10.1038/ncb0309-228 19255566

[B28] Grishok A Sinskey JL Sharp PA Transcriptional silencing of a transgene by RNAi in the soma of C. elegans Genes Dev 2005 19 6 683 696 10.1101/gad.1247705 15741313 PMC1065722

[B29] Baulcombe D RNA silencing in plants Nature 2004 431 7006 356 363 10.1038/nature02874 15372043

[B30] Lu R Maduro M Li F Li HW Broitman-Maduro G Li WX Ding SW Animal virus replication and RNAi-mediated antiviral silencing in *Caenorhabditis elegans* Nature 2005 436 7053 1040 1043 10.1038/nature03870 16107851 PMC1388260

[B31] O’ Donnell KA Wentzel EA Zeller KI Dang CV Mendell JT c-Myc-regulated microRNAs modulate E2F1 expression Nature 2005 435 7043 839 843 10.1038/nature03677 15944709

[B32] Selbach M Schwanhausser B Thierfelder N Fang Z Khanin R Rajewsky N Widespread changes in protein synthesis induced by microRNAs Nature 2008 455 7209 58 63 10.1038/nature07228 18668040

[B33] Eulalio A Huntzinger E Izaurralde E Getting to the root of miRNA-mediated gene silencing Cell 2008 132 1 9 14 10.1016/j.cell.2007.12.024 18191211

[B34] Friedman JM Jones PA MicroRNAs: critical mediators of differentiation, development and disease Swiss Med Wkly 2009 139 33–34 466 472 19705306 10.4414/smw.2009.12794 PMC2854010

[B35] Esquela-Kerscher A Slack FJ Oncomirs - microRNAs with a role in cancer Nat Rev Cancer 2006 6 4 259 269 16557279 10.1038/nrc1840

[B36] Jiang Q Wang Y Hao Y Juan L Teng M Zhang X Li M Wang G Liu Y miR2Disease: a manually curated database for microRNA deregulation in human disease Nucleic Acids Res 2009 37 Database issuue D98 104 18927107 10.1093/nar/gkn714 PMC2686559

[B37] Navarro L Dunoyer P Jay F Arnold B Dharmasiri N Estelle M Voinnet O Jones JD A plant miRNA contributes to antibacterial resistance by repressing auxin signaling Science 2006 312 5772 436 439 10.1126/science.1126088 16627744

[B38] Xiao J Li Y Wang K Wen Z Li M Zhang L In silico method for systematic analysis of feature importance in microRNA-mRNA interactions BMC Bioinforma 2009 10 427 10.1186/1471-2105-10-427 PMC3087347 20015389

[B39] De Flora S Bonanni P The prevention of infection-associated cancers Carcinogenesis 2011 32 6 787 795 10.1093/carcin/bgr054 21436188 PMC3314281

[B40] Schulte LN Eulalio A Mollenkopf HJ Reinhardt R Vogel J Analysis of the host microRNA response to Salmonella uncovers the control of major cytokines by the *let-7* family EMBO J 2011 30 10 1977 1989 10.1038/emboj.2011.94 21468030 PMC3098495

[B41] Tili E Michaille JJ Cimino A Costinean S Dumitru CD Adair B Fabbri M Alder H Liu CG Calin GA Croce CM Modulation of miR-155 and miR-125b levels following lipopolysaccharide/TNF-alpha stimulation and their possible roles in regulating the response to endotoxin shock J Immunol 2007 179 8 5082 5089 17911593 10.4049/jimmunol.179.8.5082

[B42] Cremer TJ Ravneberg DH Clay CD Piper-Hunter MG Marsh CB Elton TS Gunn JS Amer A Kanneganti TD Schlesinger LS Butchar JP Tridandapandi S MiR-155 induction by *F. novicida* but not the virulent *F. tularensis* results in SHIP down-regulation and enhanced pro-inflammatory cytokine response PLoS One 2009 4 12 e8508 10.1371/journal.pone.0008508 20041145 PMC2794384

[B43] Schnitger AK Machova A Mueller RU Androulidaki A Schermer B Pasparakis M Kronke M Papadopoulou N *Listeria monocytogenes* infection in macrophages induces vacuolar-dependent host miRNA response PLoS One 2011 6 11 e27435 10.1371/journal.pone.0027435 22114673 PMC3219661

[B44] Jackson DB Serum-based microRNAs: are we blinded by potential? Proc Natl Acad Sci USA 2009 106 1 E5 10.1073/pnas.0809999106 19106287 PMC2629234

[B45] Fu Y Yi Z Wu X Li J Xu F Circulating microRNAs in patients with active pulmonary tuberculosis J Clin Microbiol 2011 49 12 4246 4251 10.1128/JCM.05459-11 21998423 PMC3232949

[B46] Yi Z Fu Y Ji R Li R Guan Z Altered micro RNA signatures in sputum of patients with active pulmonary tuberculosis PLoS One 2012 7 8 e43184 10.1371/journal.pone.0043184 22900099 PMC3416796

[B47] Sharbati J Lewin A Kutz-Lohroff B Kamal E Einspanier R Sharbati S Integrated microRNA-mRNA-analysis of human monocyte derived macrophages upon *Mycobacterium avium* subsp. *hominissuis* infection PLoS One 2011 6 5 e20258 10.1371/journal.pone.0020258 21629653 PMC3101234

[B48] Ma F Xu S Liu X Zhang Q Xu X Liu M Hua M Li N Yao H Cao X The microRNA miR-29 controls innate and adaptive immune responses to intracellular bacterial infection by targeting interferon γ Nat Immunol 2011 12 9 861 869 10.1038/ni.2073 21785411

[B49] Rajaram MV Ni B Morris JD Brooks MN Carlson TK Bakthavachalu B Schoenberg DR Torrelles JB Schlesinger LS *Mycobacterium tuberculosis* lipomannan blocks TNF biosynthesis by regulating macrophage MAPK-activated protein kinase 2 (MK2) and microRNA miR-125b Proc Natl Acad Sci USA 2011 108 42 17408 17413 10.1073/pnas.1112660108 21969554 PMC3198317

[B50] Singh Y Kaul V Mehra A Chatterjee S Tousif S Dwivedi VP Suar M Kaer LV Bishai WR Das G *Mycobacterium tuberculosis* controls miR-99b expression in infected murine dendritic cells to modulate host immunity J Biol Chem 2012 Epub ahead of print 10.1074/jbc.C112.439778 PMC3576108 23233675

[B51] Wu J Lu C Diao N Zhang S Wang S Wang F Gao Y Chen J Shao L Lu J Zhang X Weng X Wang H Zhang W Huang Y Analysis of microRNA expression profiling identifies miR-155 and miR-155* as potential diagnostic markers for active tuberculosis: a preliminary study Hum Immunol 2012 73 1 31 37 10.1016/j.humimm.2011.10.003 22037148

[B52] Kumar R Halder P Sahu SK Kumar M Kumari M Jana K Ghosh Z Sharma P Kundu M Basu J Identification of a novel role of ESAT-6-dependent miR-155 induction during infection of macrophages with Mycobacterium tuberculosis Cell Microbiol 2012 14 10 1620 1631 10.1111/j.1462-5822.2012.01827.x 22712528

[B53] Ghorpade DS Leyland R Kurowska-Stolarska M Patil SA Balaji KN MicroRNA-155 is required for *Mycobacterium bovis* BCG-mediated apoptosis of macrophages Mol Cell Bio 2012 32 12 2239 2253 10.1128/MCB.06597-11 22473996 PMC3372268

[B54] Liu Y Wang X Jiang J Cao Z Yang B Cheng X Modulation of T cell cytokine production bymiR-144* with elevated expression in patients with pulmonary tuberculosis Mol Immunol 2011 48 9–10 1084 90 21367459 10.1016/j.molimm.2011.02.001

[B55] Liu PT Wheelwright M Teles R Komisopoulou E Edfeldt K Ferguson B Mehta MD Vazirnia A Rea TH Sarno EN Graeber TG Modlin RL MicroRNA-21 targets the vitamin D-dependent antimicrobial pathway in leprosy Nat Med 2012 18 2 267 273 10.1038/nm.2584 22286305 PMC3274599

[B56] Wu Z Lu H Sheng J Li L Inductive microRNA-21 impairs anti-mycobacterial responses by targeting IL-12 and Bcl-2 FEBS Lett 2012 586 16 2459 2467 10.1016/j.febslet.2012.06.004 22710123

[B57] Kumar S Naqvi RA Khanna N Rao DN Disruption of HLA-DR raft, deregulations of Lck-ZAP-70-Cbl-b cross-talk and miR181a towards T cell hyporesponsiveness in leprosy Mol Immunol 2011 48 9–10 1178 1190 21453975 10.1016/j.molimm.2011.02.012

[B58] Cho WC OncomiRs: the discovery and progress of microRNAs in cancers Mol Cancer 2007 6 60 10.1186/1476-4598-6-60 17894887 PMC2098778

[B59] Zen K Zhang CY Circulating microRNAs: a novel class of biomarkers to diagnose and monitor human cancers Med Res Rev 2012 32 2 326 348 10.1002/med.20215 22383180

[B60] Maertzdorf J Weiner J 3rd Mollenkopf HJ TB Network TBon Bauer T Prasse A Muller-Quernheim J Kaufmann SH Common patterns and disease-related signatures in tuberculosis and sarcoidosis Proc Natl Acad Sci USA 2012 109 20 7853 7858 10.1073/pnas.1121072109 22547807 PMC3356621

[B61] O’Connell RM Rao DS Chaudhuri AA Baltimore D Physiological and pathological roles for microRNAs in the immune system Nat Rev Immunol 2010 10 2 111 122 10.1038/nri2708 20098459

[B62] Takeshita F Patrawala L Osaki M Takahashi RU Yamamoto Y Kosaka N Kawamata N Kelnar K Bader AG Brown D Ochiya T Systemic delivery of synthetic microRNA-16 inhibits the growth of metastatic prostate tumors via down regulation of multiple cell-cycle genes Mol Ther 2010 18 1 181 187 10.1038/mt.2009.207 19738602 PMC2839211

[B63] Lanford RE Hildebrandt-Eriksen ES Petri A Persson R Lindow M Munk ME Kauppinen S Orum H Therapeutic silencing of microRNA-122 in primates with chronic hepatitis C virus infection Science 2010 327 5962 198 201 10.1126/science.1178178 19965718 PMC3436126

[B64] Wang C Yang S Sun G Tang X Lu S Neyrolles O Gao Q Comparative miRNA Expression Profiles in Individuals with Latent and Active Tuberculosis PLoS One 2011 6 10 e25832 10.1371/journal.pone.0025832 22003408 PMC3189221

[B65] Guo W Li JT Pan X Wei L Wu JY Candidate *Mycobacterium tuberculosis* genes targeted by human microRNAs Protein Cell 2010 1 5 419 421 10.1007/s13238-010-0056-4 21203954 PMC4875133

